# Thermomechanical performance of continuous carbon fibre composite materials produced by a modified 3D printer

**DOI:** 10.1016/j.heliyon.2023.e13581

**Published:** 2023-02-15

**Authors:** A. Le Duigou, M. Grabow, M. Castro, R. Toumi, M. Ueda, R. Matsuzaki, Y. Hirano, J. Dirrenberger, F. Scarpa, R. D'Elia, K. Labstie, U. Lafont

**Affiliations:** a Université de Bretagne Sud, IRDL UMR CNRS 6027, Bionics Group, Centre de recherche C Huygens, 56100 Lorient, France; b Nihon University, 1-8-14 Kanda-surugadai, Chiyoda, Tokyo 101-8308, Japan; c Tokyo University of Science, 2641 Yamazaki, Noda, Chiba 278-8510, Japan; d Japan Aerospace Exploration Agency, 6-13-1 Osawa, Mitaka, Tokyo, 181-0015, Japan; e Laboratoire PIMM, Arts et Métiers-ParisTech, CNAM, CNRS, 75013 Paris, France; f Aerospace Engineering, Bristol Composites Institute, School of Civil, Aerospace and Mechanical Engineering, University of Bristol, BS8 1TR, UK; g ICA-Mines d’Albi, Campus Jarlard 81013 Albi CT Cedex 09, France; h IRT Saint-Exupéry, 31405 Toulouse, France; i European Space Research and Technology Centre, European Space Agency, Keplerlaan 1, 2201 AZ Noordwijk, the Netherlands

**Keywords:** Composite, 3D printing, Mechanical properties

## Abstract

First of all, this article aimed to evidence the role of a modified printer developed for continuous carbon fibre reinforced PolyAmide (cCF/PA6-I) together with the use of a fully open slicing step on the printing quality and the longitudinal/transverse tensile and in-plane shear properties. A comprehensive assessment of the microstructure and properties with a similar material (cCF/PA6-I), but produced with a commercial printer (i.e., Markforged® MarkTwo) has been achieved. Our customised printer and the open slicer used have made possible to better control the print conditions (i.e., layer height and distance between filaments), to reduce the porosity from more than 10% to about 2% and improve the mechanical properties.

Moreover, the understanding of the behaviour of these 3D printed composites with wide-ranging external temperatures is mandatory for future use in a severe environment and/or development of new thermally active 4D printed composites.

The 3D printed cCF/PA6-I composites have been then thermomechanically characterised along different printing directions (0, 90 and ± 45°) from −55 to +100 °C. Unlike the longitudinal properties that hardly change with temperature, the transverse and in-plane shear stiffness and strength of these 3D printed composites were particularly sensitive to temperature variations, with decreases of 25–30% and 30–55%, respectively. This was due to the high sensitivity of the polymer matrix, the fibre/matrix and interfilament interfaces when the composites were loaded along those directions, because damages induced by internal thermal stresses. Fractography has also been carried out to reveal damage mechanisms.

## Graphical abstract

**Figure:**
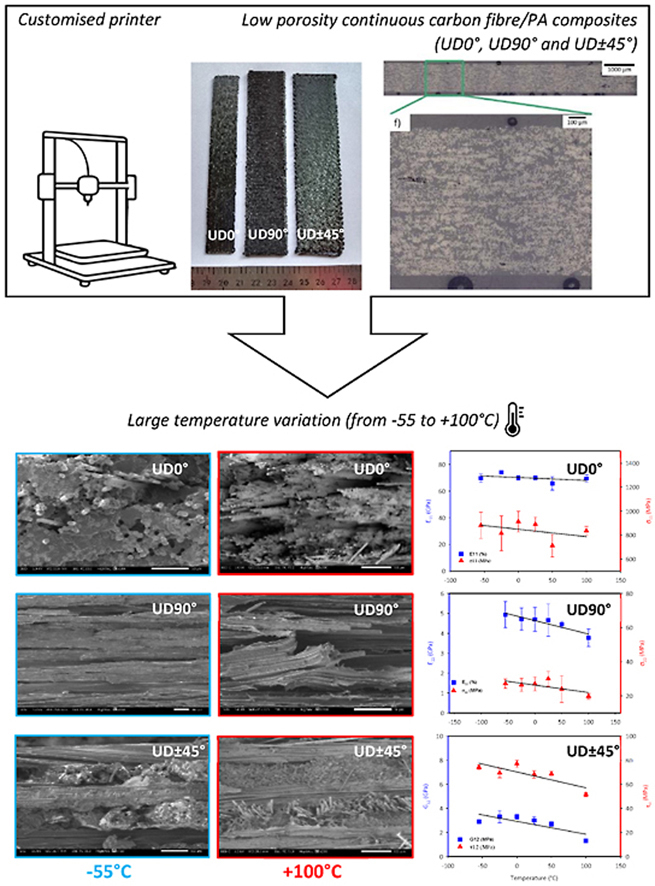

## 1 Introduction

Continuous carbon fibre reinforced polymer composites (CFRPC) are widely used in the field of transport, marine, aerospace, and space applications due to their high specific mechanical properties compared to metal alloys.

The application of 3D printing to CFRPs opened a new era for the design and fabrication of complex composite structures, for which cost and environmental impact can be tailored by the amount of material [1]. It is now widely recognized that additive manufacturing of polymer materials is a major tool to meet the challenges provided by extreme operational environments, such as those present in space exploration, by proposing a new generation of materials [1]. For example, NASA's Made In Space [2] has managed to realise the world's first experiment on 3D printing in space on the international space station in 2014. In 2018, NASA also introduced the ‘Refabricator’ concept, which can turn waste back into raw materials for re-printing. In 2017, ESA (European Space Agency) fabricated 3D printed CubeSat structures, incorporating their own electrical lines. These can be printed using PEEK and other high thermoplastic grades, as they are recyclable and biocompatible [3]. A further step towards high mechanical performances of 3D printed composites can now be achieved by using the pre-impregnated continuous carbon fibre printing strategy [4]. Tian et al. [5] and Matsuzaki et al. [6] were among the first to develop a machine for 3D printing of Carbon fibre reinforced polymer composites. In 2015, Markforged® (USA) began selling the MarkOne, the world's first commercially available 3D printer that produced continuous fibre reinforced composites (CFRPC). Since then, this printer has been widely used for CFRPC 3D printing research, although it is now being challenged by new brands (Anisoprint®, 9T Labs® …). From 2016, investigators from JAXA (Japan Aerospace Exploration Agency) are involved in research dedicated to 3D printing of continuous fibre composites [6]. Then, applications of the 3D printed CFRPC concept have been investigated. In 2020, for example, CAST (China Academy of Space Technology) completed the first Chinese 3D printing experiment in a spacecraft using continuous carbon fiber reinforced PLA composites [4].

However, mechanical performances of CFRPC were still below those of conventional composites due to the presence of printing defects provided lack of slicing step control: inter/intra filament voids [7,8], fibre breakage at tight curvatures [9] and to the lack of control of the distance between filaments and layer height. In addition, their evolution in response to environmental changes (temperature or moisture) is still not well understood. Chabaud et al. [10] have evaluated the effects of the relative humidity (10–98% RH) on sorption, hygroexpansion and mechanical properties of continuous carbon fibre reinforced PA composites. At 98% RH, a non-negligible moisture absorption was observed, resulting in a reduction of PA/carbon stiffness and strength by 25% and 18% in the longitudinal and 45% and 70%, respectively, in the transverse direction.

First of all, this article aims to evidence the role of a modified fused filament fabrication printer developed for continuous carbon fibre reinforced PA (cCF/PA6-I from Markforged®) together with the use of a fully open slicing step on the printing quality and the longitudinal tensile properties. Comparison will be achieved with a similar material but produced by one of the most well-known 3D printers for continuous fibre composites, Markforged® Marktwo.

Then, deeper insight will be provided about the evolution of the mechanical behaviour and properties of 3D printed cCF/PA6-I by customised printer along the longitudinal (0°), transverse (90°) and in-plane shear (±45°) directions when subjected to high temperature variations (temperature range from −55 to +100 °C). The effect of the temperature on the damage mechanism is finally discussed using fractography obtained by SEM observations.

## 2 Materials and methods

### 2.1 Materials

The composite filament (carbon fibre, Markforged®) consists of continuous carbon fibres (vf = 35%) embedded in a polyhexamethylene-isophtalamide matrix (cCF/PA6-I). The glass transition temperature of the PA 6-I matrix is 125 ± 2.5 °C. The cCF/PA6-I filaments have a diameter of 379,8 ± 10,5 μm, while the porosity volume in the filament prior to printing was measured to be 1.85 ± 0.3% [7]. The average coefficient of thermal expansion measured by dynamic mechanical analysis (Q800 DMA from TA Instruments) over a temperature range from −50 to 200 °C is 50 10^−6^ °C^−1^ for transversely and assumed to be negligible for longitudinally oriented cCF/PA6-I due carbon fibre properties.

### 2.2 3D printers and manufacturing parameters

For this study a custom-made 3D printer is used. The printing quality and induced mechanical performance are compared to similar cCF/PA6-I materials printed with a commercial MarkTwo from Markforged®.

**Fig. 1:**
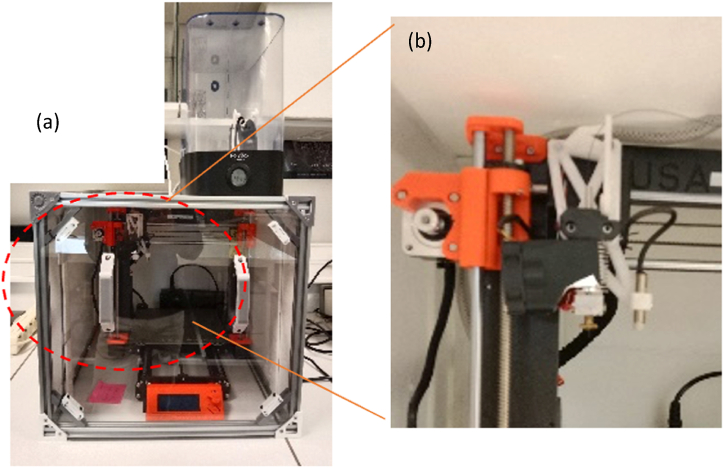
(a) Image of the modified printer for continuous carbon fibre composite printing. A humidity-controlled box is present, in which the feeder guides the filament into a thermally isolated chamber. (b) The zoomed image on the right shows the print head with Pinda sensor system and customised brass nozzle.

The in-house designed and custom-made 3D printer is based on a Prusa MK3s (Fig. 1a and b) and inspired by facilities described in Refs. [12,13]. This 3D printer has a Bowden-like extruder with a roller to feed the continuous cCF/PA6-I filament and to reduce sudden pull when the nozzle pulls the filament. Thus, a better print accuracy can be achieved, especially at the edge of the samples. A thin polytetrafluoroethylene (PTFE) tube with an inner diameter of 2 mm was attached to the feeder drive to guide the filament directly into the nozzle assembly. An original brass nozzle with an inner diameter of 0.8 mm was mounted to the heating block in order to find a compromise between avoiding clogging and improving print accuracy. The heater has been also modified to allow printing at high temperatures while using a flat nozzle. The layer height is controlled by the Pinda sensor of the Prusa system. To ensure high print quality, the cCF/PA6-I spool has been placed in a humidity-controlled box where the filament is pulled through a close circuit until it reaches a box containing the printer. An isolated chamber is also used to reduce heat losses.

Table 1 gathers the slicing and printing parameters used on the custom printer compared to those used on Marktwo from Markforged®. Unlike Eiger® slicing software (designed by MarkForged) that were used with the commercial printer, an open-source software for unconstrained design in additive manufacturing, FullControl Gcode Designer [11], is used with the custom printer.

**Table 1:** Printing and slicing parameters used with MarkTwo from Markforged® and those used from the custom Prusa printer.

	MarkTwo from Markforged ® [2]	Modified Prusa (present work)
Interfilament distance (mm)	X (filling 100%)	1.2
Layer height (mm)	0.125	0.1
T nozzle	255	280
T bed	Not heated	90
Printing speed (mm/s)	14–15	10

Prior to printing, cCF/PA6-I filaments were conditioned in a vacuum heat chamber at 60 °C for 72 h to remove sorbed moisture and thus ensure high print quality. After manufacturing, all printed parts (sample) stored in a chamber under vacuum before being tested.

### 2.3 Material characterisation

#### 2.3.1 Microanalysis

In the context of Fused Filament fabrication of continuous fibre composites, the print quality from commercially available printers is questionable [7]. Thus, controlling the print quality prior to characterisation is a mandatory step to develop robust and reliable manufacturing processes and print structures.

A microstructure analysis has been performed to determine the porosity content of the printed material. The samples have been polished and placed under a microscope (Keyence VHX-7000) with different magnifications (×200 & ×500). The content of porosity has been measured from the acquired images using the ImageJ software. Five pictures have been used to evaluate each parameter for the pristine and printed material. Validation has also been carried out by gravimetric measurements in ethanol according to the ASTM D792 standard.

#### 2.3.2 Thermomechanical characterisation

Tensile tests have been performed using an INSTRON 500 kN machine on samples made of cCF/PA6-I with three different orientations to their load axis (0°, 90° and ±45°). The samples have been designated as UD0°, UD90° and ±45°. The specimens have been printed with three different LxWxT dimensions: 120 × 15 × 1 mm^3^ for UD0°, 120 × 25 × 2 mm^3^ for UD90° and 120 × 30 × 1.5 mm^3^ for ±45°.

Prior to testing, the edges (serpentine filament) of the UD90° and ±45° samples have been machine-milled and carefully polished to reduce uncertainties in the subsequent determination of the mechanical behaviour and properties. It has been previously shown how the edge effect with continuous fibre reinforced filaments affects the mechanical behaviour and properties [14]. End tabs have then been glued with heat resistant adhesive on all samples and strain gauges glued in the centre of UD0° and ±45° samples. The specimens have been wired and put under vacuum until the testing of the samples has been completed. The speed of the cross-bar was 2 mm/min. The strain in the UD90° samples has been measured with an extensometer and for UD0° and ±45° samples with strain gauges. Using an environmental chamber attached to the tensile test machine, tests have been made at six different temperatures: 50 °C, −25 °C, 0 °C, 25 °C, 50 °C and 100 °C. A minimum of five samples per batch have been tested.

#### 2.3.3 Fractography

The cCF/PA6-I samples fractured after the tensile tests at −55 °C and +100 °C have been examined with a scanning electron microscope SEM (JEOL JSM-IT500HR) to compare the microstructures of the samples exposed from low to high temperatures. Sections from the fractured surface of the samples have been cut and then coated with a thin layer of gold using an Edwards sputter coater.

## 3 Results and interpretation

### 3.1 Print quality

**Fig. 2:**
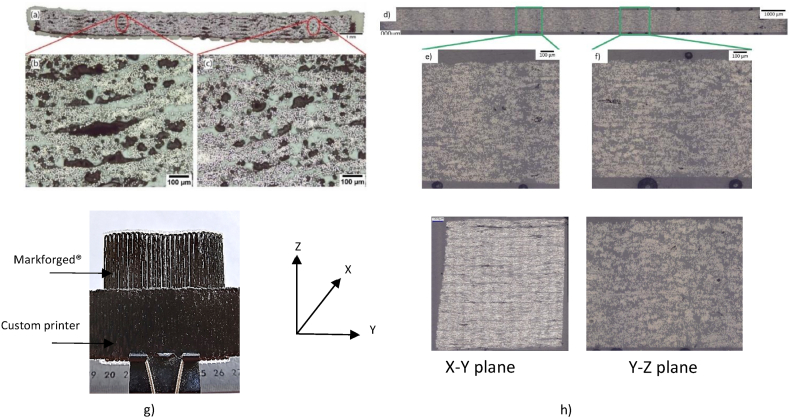
Cross section of the cCF/PA6-I material printed with (a, b and c) the Markforged® 3D print device [7] and with (d, e and f) the customized printer for continuous carbon fibre composite printing. (g) X–Y plane of cCF/PA6-I material printed with Markforged® and custom printer highlighting the difference of distance between filament, (h) Cross sections of the X–Y and Y–Z planes (customised printer).

Prior to mechanical characterisation, the printed cCF/PA6-I samples have been qualified with previously optimised printing/slicing parameters (temperature nozzle and bed, layer height, interfilament distance, printing speed) and their microstructure observed. First, a comparison can be made between the cCF/PA6-I printed samples with a commercial MarkTwo Markforged® printer (Fig. 2a, b and c) and the same material but printed with the customised printer (Fig. 2 d, e and f). One ply cCF/PA6-I samples printed either with Markforged® or custom printer are macroscopically observed in the X–Y plane (Fig. 2g).

A heterogenous microstructure is observed for samples printed with Markforged® device with larger induced interfilament distance (no controlled, see Table 1) compared to those printed with customized printer (ID = 1.2 mm).

Sectioned and polished cross sections (X–Y and Y–Z planes) of cCF/PA6-I samples printed with the customised print device are also investigated to fully evaluate the defect distribution within an apparent material volume (Fig. 2h).

Image analysis and gravimetric measurement revealed a porosity content of 2.0 ± 0.7% for the X–Y plane, and 1.2 ± 0.7% for the Y–Z plane compared to a globally porosity content of 15.1 ± 0.3% observed in samples printed with the commercial printer [7]. Using the commercial Markforged® printer leads to both inter-filament and interply porosity. Indeed, the Mark Two® printer and Eiger® do not give the same flexibility compared those used in the present work. Indeed, several printing and slicing parameters such as the extrusion and bed temperatures are constant and cannot be modified (Table 1). However, nozzle temperature influences the interdiffusion mechanism ability of the polymer and thus the off-axis properties. In addition, the bed, is not heated which implies several issues like thermal residual stresses, lack of adhesion …

Another fundamental slicing parameter is not directly controlled: the interfilament distance (ID). Eiger® interface only allows a percentage of infill to be set. For example, the printed cCF/PA6-I samples were printed with 100% infill and imply lack of overlapping between filaments. The interdiffusion of the polymer chains that generates the adhesion between the filaments is actually a function of the pressure applied in the molten state. Consequently, during off-axes testing (UD90° and UD ± 45°), the loads are not transferred properly to the reinforcing fibres and adhesive rupture between two adjacent beads happens [3].

With the custom printer, the porosities are significantly reduced by freely optimising the printing/slicing parameters, especially the layer height and the interfilament distance (ID = 1.2 mm). Hence, the interdiffusion of the polymer chains between adjacent filament is triggered thanks to the pressure applied in the molten state. Fracture analysis (Table 4) evidenced for UD90° and UD ± 45° that interfilament failure is not observed, only fibre/matrix interfacial failure and matrix failure are noticed.

Within, the custom printed sample, the porosities are also mainly located in the interfilament area (X–Y plane in Fig. 2h) as lateral pressure applied by the by the nozzle is more difficult than exerting vertical pressure on a previous layer (Y–Z plane).

**Fig. 3:**
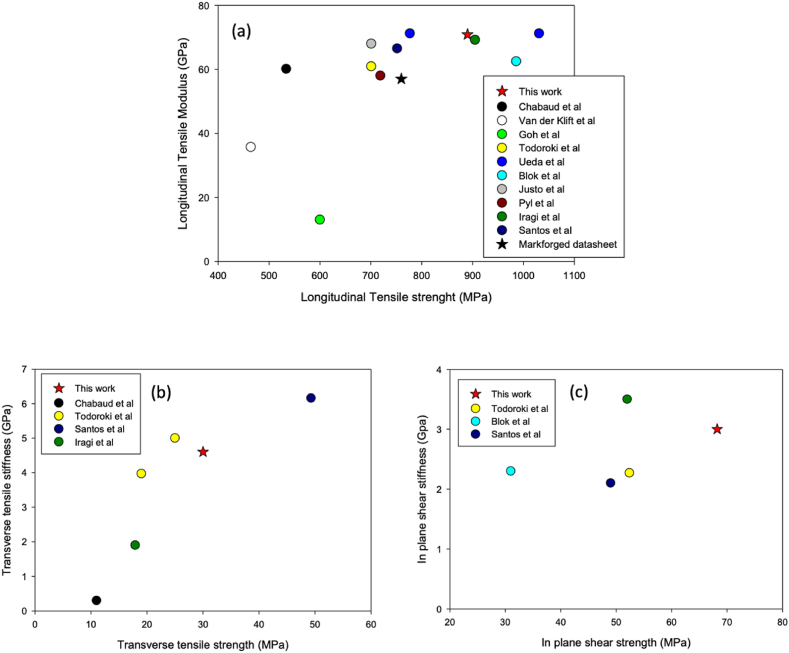
Summary of the mechanical performances of 3D printed cCF/PA6-I material, provided by Markforged®. (a) Shows the longitudinal tensile, (b) the transverse tensile and (c) the in-plane shear strengths. The samples have been printed using customised print devices (in the present work and in the work of Ueda et al. [15]) or commercial printers, such as MarkOne or MarkTwo by Markforged® (works of [7,8,[14], [15], [16], [17], [18], [19], [20], [21]]).

Fig. 3a–c present the overall tensile performance (longitudinal tensile, transverse tensile and in plane shear strength) of cCF/PA6-I samples obtained at 20 °C. The samples produced here are compared to those presented in open literature using the same material but Markforged® or other customised printers [15].

Even assuming that the printed material is the same and the printing strategy similar (10% infill), a large scatter of the mechanical properties is evident. Several explanations can be proposed for this:

- When the Markforged® printer is used together with the Eiger® slicer, the bottom and top layers, as well as the outer periphery of each layer are always printed with the 100% ± 45° infill Nylon filament [8]. This has a negative effect because it reduces the overall fibre volume fraction for the part [7] and makes further comparison difficult. The effect is more important on the transverse and in-plane shear properties [17]. Also, the number of Nylon layers (wall or roof) effectively used is not always well described in the available articles.
- The shape of the sample that can produce stress concentrations with premature failure.
- Some authors have cut the edges of samples as they contain serpentine folded fibre bundles (present work [14,19]). Todoroki et al. [14] have demonstrated that the transverse elastic modulus and the transverse strength can be 25% higher when serpentine folded fibre bundles are present, even if only a small part of the fibre filaments work parallel to the applied load during the transverse tensile test. In contrast, the shear properties decrease by a factor of two when serpentine folded fibre zones are cut.
- Samples printed with customised printers under carefully controlled slicing parameters and environmental conditions (e.g. relative humidity) show larger overall longitudinal and transverse tensile stiffness and strengths, and in-plane stiffness and strength compared to specimens produced using commercial printers (Fig. 3a–c). For example, the samples produced within the present work exhibit drastically larger properties (+15% in E_11_, +33% in σ_11_; +1500% in E_22_, +100% in σ_22_) than those obtained in our laboratory by Chabaud et al. [7] with the same material but printed with a commercial device. Indeed, the non-optimised parameters of layer height and interfilament distance, but also the presence of moisture, can lead to a higher porosity content close to 10% for all the cited references; the porosity does not exceed 2% in the present work.

Some references also show very high mechanical performances, e.g. Iragi et al. [19] and Blok et al. [8] for longitudinal, and Santos et al. [17] for the transverse tensile properties. There is no clear explanation as to how they managed to exceed the performance given by the Markforged® datasheet (+20% in tensile strength for Blok et al.) with similar materials and printer.

Finally, mechanical properties that can compete with those from similar composites with same fibre content and conventional manufacturing can be achieved using a customised printer [22]. Further improvements can be obtained by increasing the fibre content, the temperature range and the pressure application [4]. For example, Ueda et al. [15] (blue dots in Fig. 3a) have developed a roller compaction that increases the tensile strength of cCF/PA6-I by almost 30%.

### 3.2 Thermomechanical characterisation

#### 3.2.1 Thermomechanical behaviour

After printing and storage in a humidity-controlled chamber (10% relative humidity) to prevent moisture absorption, the cCF/PA6-I samples with different fibre orientations (0°, 90° and ±45°) are thermomechanically characterised in a heat chamber ranging from −55 °C to +100 °C.

**Fig. 4:**
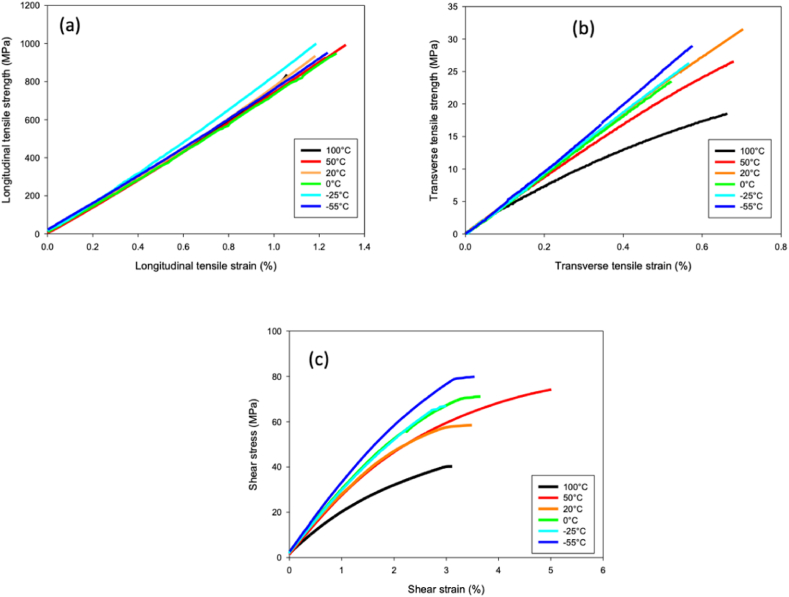
(a) Longitudinal and (b) transverse tensile strengths, as well as in-plane shear behaviour of 3D printed cCF/PA6-I samples tested in a temperature range from −55 to +100 °C.

The printed laminates with different reinforcement orientations allow a better understanding of the physical effects provided by an environment with strong temperature fluctuations. In addition, they can serve as a basis for the design and programming of a 4D printing process based on thermal actuation [23]. Fig. 4a–c present the longitudinal and transverse tensile strength, as well as the in-plane shear behaviour of cCF/PA6-I samples within a temperature range from −55 to +100 °C.

The large temperature variation from −55 to +100 °C has no effect on the longitudinal tensile behaviour of cCF/PA6-I, which remains linear-elastic and shows a similar trend towards stiffness and brittleness as at 20 °C. This is due to the fact that the longitudinal mechanical behaviour is mainly dominated by the behaviour of the fibres, while the latter (i.e., the carbon fibres) are highly anisotropic and relatively insensitive to the given temperature range.

Unlike the longitudinal tensile behaviour of the composite, the transverse tensile strength and the in-plane shear properties further depend on the matrix and the bond strength of the fibre/matrix interface [24]. Therefore, the behaviour of the corresponding cCF/PA6-I composites is logically altered by temperature changes, especially when the ambient temperature approaches the glass transition temperature (Tg) of the polymer matrix [7]. Other researchers have highlighted that a Tg of 122 °C corresponds to the one of PA6/3T [25]. Hence, the T_g_–T_test_ difference is a main parameter that controls the thermomechanical behaviour of thermoplastic composites along the transverse and shear directions.

From a stiff and almost elastic-linear behaviour at low and moderate positive temperatures (−55 °C–50 °C), the transverse tensile and shear behaviour of cCF/PA6-I becomes then more fragile and non-linear with a pronounced loss of linearity, probably due to internal damages.

#### 3.2.2 Thermomechanical properties

**Fig. 5:**
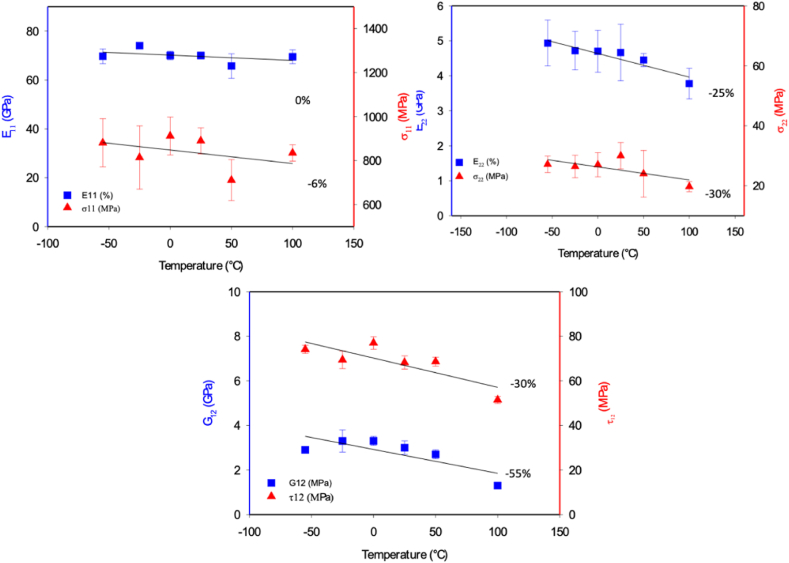
(A) Longitudinal tensile, (b) transverse tensile, and (c) in-plane shear stiffness and strengths of cCF/PA6-I in a temperature range from −55 to +100 °C.

He longitudinal and transverse tensile stiffness and strengths E_11_, σ_11_; E_22_, σ_22_ and in-plane shear stiffness and strength G_12_, τ_12_ are shown in Fig. 5a–c, respectively. These numerical data are also reported in Table 2.

**Table 2:** Mechanical properties of 3D printed cCF/PA6I samples, printed using a customised printer, in a temperature range from-55 to +100 °C.

	$E_{11}$ (GPa)	$\sigma_{11}$ (MPa)	$\varepsilon_{11}$ (%)	$E_{22}$ (GPa)	$\sigma_{22}$ (MPa)	$\varepsilon_{22}$ (%)	$G_{12}\left(GPa\right)$	$\tau_{12}\;\left(MPa\right)$	$\gamma_{12}$ (%)
100 °C	69.5 ± 2.8	834.8 ± 37.3	0.8 ± 0.2	3.8 ± 0.4	19.7 ± 1.7	0.8 ± 0.2	1.6 ± 0.4	51.3 ± 3.7	3.4 ± 0.5
50 °C	65.7 ± 5.0	711.0 ± 93.0	0.9 ± 0.3	4.4 ± 0.2	24.0 ± 7.8	0.9 ± 0.3	2.7 ± 0.1	74.9 ± 6.5	5.0 ± 0.2
23 °C	70.9 ± 1.6	890.1 ± 87.7	1.2 ± 0.1	4.6 ± 0.8	30 .0 ± 4.3	1.5 ± 0.7	3.0 ± 0.2	68.2 ± 6.5	3.3 ± 0.5
0 °C	69.8 ± 4.2	963.5 ± 70.0	1.3 ± 0.1	4.7 ± 0.6	27 .2 ± 3.1	1.0 ± 0.3	3.3 ± 0.2	84.1 ± 3.1	3.3 ± 0.4
−25 °C	74.2 ± 1.2	951.9 ± 42.7	1.1 ± 0.2	4.7 ± 0.5	26.4 ± 3.8	0.5 ± 0.1	3.3 ± 0.2	75.6 ± 9.6	3.4 ± 0.2
−55 °C	71.4 ± 7.1	991.3 ± 82.2	1.2 ± 0.1	4.9 ± 0.6	27.2 ± 2.7	0.5 ± 0.1	2.8 ± 0.1	74.1 ± 1.9	3.8 ± 0.8

The tensile strength in the longitudinal direction is hardly affected by the temperature. A linear decrease of 0% and 6% between −50 and +100 °C can be observed (Fig. 5a). For the same temperature range, the shear strength in the transverse direction and in the in-plane one decreases drastically with the temperature change (−25% and −30% for transverse stiffness and strength and −30 to −55% for in-plane stiffness and strength, respectively).

A fractography analysis has been performed for all laminate configurations on the macro- (Table 3) and microscale (Table 4) at −55 °C and +100 °C. At the macroscale level, the temperature does not affect the failure mechanism of the UD0° samples. The failure mode consisted in splitting crack and fibre rupture. Fibre failure occurred both within the gage length and near the gripping area. Fibre rupture near the gripping area changed the measured strength, something that has also been observed by Ueda et al. [15].

**Table 3:** Photography of the failure location of cCF/PA6-I UD0°, UD90° and UD±45° samples tested at −55 °C and +100 °C.

	−55 °C	100 °C
UD0°		
UD90°		
UD ± 45**°**

**Table 4:** SEM micrographs of cCF/PA6-I UD0°, UD90° and UD±45° samples tested at −55 °C and +100 °C. Blue and red rectangles show the zooms of different areas.

	−55 °C	100 °C
UD0°		
UD90°		
UD ± 45°		

On the microscale, a longer debonding of the carbon fibres is clearly observed in the UD0° laminates (see red square in Table 4), underlining a reduction of the interfacial bonding and of the load transfer mechanism according to the Kelly-Tyson equations [26]. At −55 °C, fibre failure is observed. The debonding length is short and some holes can be observed due to debonding. Larger debonding lengths at higher temperatures also show that the fracture of the fibre/matrix interface is the predominant mechanism occurring before the failure of the fibre reinforcement. The strength of the interface bond with a thermoplastic matrix actually depends on the residual stress state at the interface and the associated friction caused by the difference in thermal expansion between the fibre and polymer matrix [27]. After the 3D printing process and cooling, a stress state develops at the interface, which can be compressive due to the larger polymer shrinkage compared to the one provided by the fibres. An increase in temperature close to Tg then leads to internal thermal tensile stresses at the fibre/matrix interface, which balance the compressive stresses and release the residual stresses. This is confirmed by the work of Thomason et al. [28] on PP/glass microcomposites where the interfacial shear strength (IFSS) has shown a strong inverse relationship with temperature. In addition, the increase of temperature generates differential expansion between carbon fibre and the matrix that should imply interfacial shear stress and then debonding if it overcome IFSS.

A change in matrix failure from fragile to ductile is also observed, but the influence is moderate during the longitudinal tensile test (Fig. 4a).

For UD90° samples, the fracture surface is flat regardless of temperature. It is important to note that failure on the macroscale is similar to what is observed in conventionally laminated CFRPs, but different from 3D printed cCF/PA6-I composites where the filament edges are not cut [7].

Indeed, in the latter case, debonding between filaments is a predominant failure mechanism due to lack of adhesion during the printing process. This has been discussed earlier and can be explained by non-optimised slicing parameters of the conventional printer. In the present work, the dominant transverse failure or weakest link is usually at the fibre/matrix interface and not in the interfilament area. This confirms the print quality achieved by using the customised printer. On the microscale, an increase in temperature from −55 to 100 °C shows a transition from fragile to ductile failure with plastic polymer deformation. Bared carbon fibres (without polymer residues) are observed on UD90° samples, indicating interfacial adhesive failure and moderate interfacial adhesion. The behaviour of UD90° samples is generally governed by the matrix and the fibre/matrix interface, whose properties are largely temperature dependent. Thus, the change in tensile behaviour is explained by the deformation of the matrix and the evolution of the IFSS implied by the increase in temperature. In-plane shear samples (Table 3) show an almost flat failure at low temperature (−55 °C) which changes to a V-shape failure at high temperature (+100 °C). Furthermore, the overall shape of the specimen is drastically changed at high temperature and high applied strain with a necking process that happens.

This might be due to the high contribution provided by the matrix to the shear behaviour, while its properties depend drastically on the temperature. This is particularly emphasised by the proximity of the Tg, where the properties of the matrix should drop. On the microscale, the failure mechanism of ±45° samples shows a longer fibre debonding length with increasing temperature. The thermomechanical behaviour of ±45° samples is also governed by the fibre/matrix interface, whose properties are strongly temperature dependent. Thus, the change in tensile behaviour is explained by the deformation of the matrix and the evolution of the IFSS implied by an increase in temperature.

## 4 Conclusion

Continuous carbon fibre-reinforced polymer composites are used as substitutes of metals in many technical fields. Complex, anisotropic materials offer further possibilities for improved and customised functions. The application of 3D printing on CFRPCs is therefore of great importance to successfully design and manufacture such complex materials. Although commercial 3D printers allow the fabrication of continuous carbon fibre composites, further optimization of printing/slicing parameters are required for load-bearing applications. Moreover, the evolution of 3D printed composites with environmental variations (temperature or moisture) is still not well understood, despite their importance for their end-user applications.

The purpose of this article was to first compare the mechanical performance of a 3D printed carbon fibre reinforced PA (cCF/PA6-I), produced under controlled conditions (temperature and humidity) using a customised 3D printer, with the performance of the same material printed with a commercial printer (Markforged® MarkTwo). Then, the effects of large temperature variations (from −55 to +100 °C) on the evolution of the mechanical behaviour and properties in longitudinal, transverse and in plane shear directions was investigated.

The development of a customised printer and slicer made it possible to control the print conditions (i.e., layer height and distance between filaments), and thus reduce the porosity from more than 10% to about 2% by using image data analysis. This microstructure control is a mandatory step to obtain high quality print results and apply more complex experimental investigations or numerical simulations on 3D printed CFRPCs. More elaborated customization processes could even be proposed with an in-situ compaction system.

The temperature variation from −55 °C to 100 °C showed little change in terms of longitudinal properties and failure mechanism due to the large contribution of carbon fibres along the load direction. Transverse and in-plane shear behaviour and properties were drastically dependent on temperature due to the large contribution of the matrix and the fibre/matrix interface. Interestingly, no interfilament failure was observed, indicating good print quality. The longer length of fibre debonding with increasing temperature observed in ±45° and UD0° samples was explained by the reduction in interfacial shear strength due to the release of compressive residual stresses and damage caused by internal stresses.

## Author contribution statement

A. Le Duigou: Conceived and designed the experiments; Analyzed and interpreted the data; Contributed reagents, materials, analysis tools or data; Wrote the paper.

M. Grabow: Performed the experiments; Analyzed and interpreted the data; Wrote the paper.

M. Castro, Y Hirano, J. Dirrenberger, F. Scarpa, R D'Elia, K. Labstie, U. Lafont: Analyzed and interpreted the data; Wrote the paper.

R Toumi: Performed the experiments.

M. Ueda, R Matsuzaki: Analyzed and interpreted the data; Contributed reagents, materials, analysis tools or data; Wrote the paper.

## Funding statement

The authors would like to thank IRT Saint Exupery, the 10.13039/501100000844European Space Agency (10.13039/501100000844ESA contract 4000133620) for financial support. Antoine le Duigou wish also thanks the French Ambassady in Japan and the PHC SAKURA program.

## Data availability statement

Data will be made available on request.

## Declaration of interest's statement

The authors declare no conflict of interest.

## References

[1] Mitchell A., Lafont U., Hołyńska M., Semprimoschnig C. (2018). Additive manufacturing – a review of 4D printing and future applications. Addit. Manuf..

[2] NASA: The road to realizing in-space manufacturing. Https://NtrsNasaGov/SearchJsp?R=201400087602020.

[3] ESA. 3D Printing CubeSat Bodies for Cheaper, Faster Missions. Http://WwwEsaInt/Our_Activities/Space_Engineering_Technology/3D_printing_CubeSat_bodies_for_cheaper_faster_missions_2017.

[4] Tian X., Todoroki A., Liu T., Wu L., Hou Z., Ueda M. (2022). 3D printing of continuous fiber reinforced polymer composites: development, application, and prospective. Chin. J. Mech. Eng.: Addit. Manuf. Front..

[5] Tian X., Tang C., Cao Y. (2014).

[6] Matsuzaki R., Ueda M., Namiki M., Jeong T.-K., Asahara H., Horiguchi K. (2016). Three-dimensional printing of continuous-fiber composites by in-nozzle impregnation. Sci. Rep..

[7] Chabaud G., Castro M., Denoual C., le Duigou A. (2019). Hygromechanical properties of 3D printed continuous carbon and glass fibre reinforced polyamide composite for outdoor structural applications. Addit. Manuf..

[8] Blok L.G., Longana M.L., Yu H., Woods B.K.S. (2018). An investigation into 3D printing of fibre reinforced thermoplastic composites. Addit. Manuf..

[9] Shiratori H., Todoroki A., Ueda M., Matsuzaki R., Hirano Y. (2020). Mechanism of folding a fiber bundle in the curved section of 3D printed carbon fiber reinforced plastics. Adv. Compos. Mater..

[10] Chabaud G., Castro M., Denoual C., le Duigou A. (2019). Hygromechanical properties of 3D printed continuous carbon and glass fibre reinforced polyamide composite for outdoor structural applications. Addit. Manuf..

[11] Gleadall A. (2021). FullControl GCode Designer: open-source software for unconstrained design in additive manufacturing. Addit. Manuf..

[12] Parker M., Inthavong A., Law E., Waddell S., Ezeokeke N., Matsuzaki R. (2022). 3D printing of continuous carbon fiber reinforced polyphenylene sulfide: exploring printability and importance of fiber volume fraction. Addit. Manuf..

[13] le Duigou A., Barbé A., Guillou E., Castro M. (2019). 3D printing of continuous flax fibre reinforced biocomposites for structural applications. Mater. Des..

[14] Todoroki A., Oasada T., Mizutani Y., Suzuki Y., Ueda M., Matsuzaki R. (2020). Tensile property evaluations of 3D printed continuous carbon fiber reinforced thermoplastic composites. Adv. Compos. Mater..

[15] Ueda M., Kishimoto S., Yamawaki M., Matsuzaki R., Todoroki A., Hirano Y. (2020). 3D compaction printing of a continuous carbon fiber reinforced thermoplastic. Compos. Part A Appl. Sci. Manuf..

[16] Justo J., Távara L., García-Guzmán L., París F. (2018). Characterization of 3D printed long fibre reinforced composites. Compos. Struct..

[17] Santos J.D., Fernández A., Ripoll L., Blanco N. (2022). Experimental characterization and analysis of the in-plane elastic properties and interlaminar fracture toughness of a 3D-printed continuous carbon fiber-reinforced composite. Polymers.

[18] Pyl L., Kalteremidou K.-A., van Hemelrijck D. (2018). Exploration of specimen geometry and tab configuration for tensile testing exploiting the potential of 3D printing freeform shape continuous carbon fibre-reinforced nylon matrix composites. Polym. Test..

[19] Iragi M., Pascual-González C., Esnaola A., Lopes C.S., Aretxabaleta L. (2019). Ply and interlaminar behaviours of 3D printed continuous carbon fibre-reinforced thermoplastic laminates; effects of processing conditions and microstructure. Addit. Manuf..

[20] Goh G.D., Dikshit V., Nagalingam A.P., Goh G.L., Agarwala S., Sing S.L., Wei J., Yeong W.Y. (2018). Characterization of mechanical properties and fracture mode of additively manufactured carbon fiber and glass fiber reinforced thermoplastics. Mater. Des..

[21] van der Klift F., Koga Y., Todoroki A., Ueda M., Hirano Y., Matsuzaki R. (2016). 3D printing of continuous carbon fibre reinforced thermo-plastic (CFRTP) tensile test specimens. Open J. Compos. Mater..

[22] le Duigou A., Chabaud G., Castro M. (2021).

[23] Wang Q., Tian X., Huang L., Li D., Malakhov A.V., Polilov A.N. (2018). Programmable morphing composites with embedded continuous fibers by 4D printing. Mater. Des..

[24] Yang L., Thomason J.L. (2010). Interface strength in glass fibre–polypropylene measured using the fibre pull-out and microbond methods. Compos. Part A Appl. Sci. Manuf..

[25] Pascual-González C., Iragi M., Fernández A., Fernández-Blázquez J.P., Aretxabaleta L., Lopes C.S. (2020). An approach to analyse the factors behind the micromechanical response of 3D-printed composites. Compos. B Eng..

[26] Kelly A., Tyson W.R. (1965). Tensile properties of fibre-reinforced metals: copper/tungsten and copper/molybdenum. J. Mech. Phys. Solid..

[27] Thomason J.L., Yang L. (2014). Temperature dependence of the interfacial shear strength in glass–fibre epoxy composites. Compos. Sci. Technol..

[28] Thomason J.L., Yang L. (2011). Temperature dependence of the interfacial shear strength in glass–fibre polypropylene composites. Compos. Sci. Technol..

